# Preclinical evidence of a direct pro-survival role of arginine deprivation in multiple myeloma

**DOI:** 10.3389/fonc.2022.968208

**Published:** 2022-09-08

**Authors:** Matteo Trudu, Laura Oliva, Ugo Orfanelli, Alessandra Romano, Francesco Di Raimondo, Francesca Sanvito, Maurilio Ponzoni, Simone Cenci

**Affiliations:** ^1^ Age Related Diseases, Division of Genetics and Cell Biology, San Raffaele Scientific Institute, Milano, Italy; ^2^ University Vita-Salute San Raffaele, Milano, Italy; ^3^ Department of Surgery and Medical Specialties, University of Catania, Catania, Italy; ^4^ Pathology Unit, San Raffaele Scientific Institute, Milano, Italy

**Keywords:** AKT, arginine, autophagy, mammalian target of rapamycin, multiple myeloma, plasma cell, stress, survival

## Abstract

Multiple myeloma grows by establishing multiple interactions with bone marrow cells. These include expansion of myeloid-derived suppressor cells, which drive immunoevasion *via* mechanisms that include arginase-1-driven depletion of L-arginine, thus indirectly promoting myeloma cell survival and tumor progression. The peculiar biology of malignant plasma cells postulates that arginine depletion may benefit their fitness also directly, *e.g.*, by engaging the integrated stress response, or by stimulating autophagy through mTORC1 inhibition. We thus investigated the direct impact of arginine deprivation on myeloma cells and challenged its pathophysiological relevance *in vitro* and *in vivo*. First, we found that partial arginine depletion spared proliferation of human multiple myeloma cells at concentrations that arrest human T cells. Next, we asked if arginine shortage activates putative adaptive pathways in myeloma cells. Low arginine failed to activate the integrated stress response, as indicated by unmodified phosphorylation of the eukaryotic initiation factor 2α, but sizably inhibited mTORC1, as revealed by reduced phosphorylation of ribosomal protein S6. Notably, depressed mTORC1 activity was not sufficient to increase autophagy, as assessed by the lysosomal digestion rate of the autophagosome-associated protein, LC3-II. Rather, it stimulated mTORC2, resulting in increased phosphatidylinositol-3 kinase-dependent AKT phosphorylation and activity, leading to heightened inhibitory phosphorylation of the pro-apoptotic BAD protein. We then tested whether arginine depletion-activated AKT may protect malignant plasma cells from cell death. Indeed, culturing myeloma cells in low arginine medium significantly reduced the apoptotic effect of the first-in-class proteasome inhibitor, bortezomib, an outcome prevented by pharmacological inhibition of AKT phosphorylation. Finally, we challenged the relevance of the identified circuit *in vivo*. To gauge the pathophysiologic relevance of low arginine to myeloma growth independently of immunoevasion, we xenotransplanted human myeloma cells subcutaneously into T cell-deficient Rag2^–/–^γc^–/–^ recipient mice and treated palpable tumor-bearing mice with the clinical-grade arginase inhibitor CB1158. Arginase inhibition significantly raised serum arginine concentration, reduced tumor growth by caliper assessment, and decreased intra-tumor AKT phosphorylation *in vivo*. Altogether, our results reveal a novel direct pro-survival effect of arginine deprivation on myeloma cells, with potential therapeutic implications.

## Introduction

Multiple myeloma (MM), the second most common hematological malignancy, is a neoplastic disorder of plasma cells (PC), which typically grow in multiple foci in the bone marrow (BM), secrete monoclonal immunoglobulins and induce fatal end-organ damage, entailing bone disease, renal failure, anemia, and hypercalcemia. MM is known to develop and progress by establishing vicious interactions within the BM multi-cellular milieu (1). Among them, MM cells induce expansion and survival of myeloid-derived suppressor cells (MDSC) (2–4), which, in turn, promote tumor growth (5). MDSCs are subdivided in granulocytic/polymorphonuclear (PMN-MDSC) and monocytic (M-MDSC), respectively resembling the phenotype and morphology of neutrophils and monocytes (6). Recent reports documented relevant roles played by MDSCs in the promotion of angiogenesis, drug resistance, metastasis, and immune suppression both in solid and hematological cancers (2, 6). However, immune suppression, mediated by induction of T cell anergy, is currently acknowledged as a chief pro-tumoral effect of MDSCs (7). The mechanisms involved include release of TGFβ and IL-10, cysteine sequestration, expression of iNOS and COX2 and of tryptophan- and arginine- degrading enzymes, indoleamine 2,3-dioxygenase (IDO) and arginase-1 (6). Arginine depletion by MDSCs leads to reduced expression of the CD3 ζ chain, resulting in impaired responsiveness and proliferation arrest of T cells, thereby hindering anti-tumor immunity (8–10). This mechanism has been implicated in MM growth by both pre-clinical observations in mouse models and clinical evidence in human disease (11–13).

Arginine is a fundamental amino acid that serves as an intermediate in the urea cycle and a precursor for protein, polyamine, creatine and nitric oxide (NO) biosynthesis (14, 15). Arginine is a conditionally essential amino acid whose synthesis depends on the expression of two key cytosolic urea cycle enzymes, argininosuccinate synthase 1 (ASS1) and argininosuccinate lyase (ASL) (16). Arginine depletion may induce a pro-survival integrated stress response (ISR) by activating the amino acid starvation sensor kinase GCN2 (general control nonderepressible 2), which in turn phosphorylates eukaryotic translation initiation factor 2 alpha (eIF2α) to decrease global protein synthesis in response to nutritional stress (17). Moreover, being arginine a powerful stimulator of mechanistic target of rapamycin complex 1 (mTORC1), its deprivation has also been shown to inhibit mTORC1 *via* interaction of CASTOR1 (cytosolic arginine sensor for mTORC1 subunit 1) with GATOR2 (GAP activity towards Rags 2) (18). A fundamental cellular metabolic hub, mTORC1 inhibits autophagy while stimulating glycolysis, lipid synthesis, mRNA translation, the pentose phosphate pathway and *de novo* pyrimidine synthesis (19). Moreover, mTORC1 inhibition can stimulate the mTORC2 complex (20) resulting in increased protein kinase B (AKT) phosphorylation and kinase activity to promote cell survival *via* mechanisms that include the inhibitory phosphorylation of the apoptotic stress-transducer Bcl-2 homology 3 (BH3)-only protein BAD (21).

Normal and malignant PCs are highly vulnerable to proteostatic stress owing to unrivaled proteosynthetic activity (22). Previous work, including ours, established that high proteosynthetic activity in the secretory pathway negatively affects MM cell fitness and is causally linked with proteasome inhibitor vulnerability (23–25). Moreover, we and others have shown that autophagy is essential to maintain proteostasis and survival of PCs (26, 27). Hence, PCs may benefit from reduced protein synthesis afforded by activation of the ISR that, while reducing global protein synthesis *via* eIF2α phosphorylation, may enhance ATF4 expression and the downstream activation of pro-survival strategies, including autophagy, antioxidant responses and amino acid metabolism (28, 29). Moreover, MM proved resistant to pharmacological inhibition of mTORC1, possibly *via* induction of the mTORC2/AKT pathway (30, 31). Notably, AKT activation has been shown to sustain tumor cell survival, tumor growth, and chemotherapeutic resistance across diverse cancers, including myeloma (32).

Building on this rationale, we set out to test the hypothesis that partial arginine starvation could exert direct beneficial effects on MM cell fitness and survival and adopted a coherent reductionist approach to define such effect, to explore the underlying mechanisms, and to gauge its pathophysiologic significance.

## Methods

### Cell cultures

Human multiple myeloma (MM) cell lines (kindly provided by Giovanni Tonon, San Raffaele Scientific Institute, Milan, Italy) and the human Jurkat T cell line were maintained in RPMI medium (Thermo Fisher Scientific, 88365) supplemented with L-Lysine (0.274 mM; Sigma-Aldrich L5751), L-Arginine (at different concentrations, as indicated; Sigma-Aldrich A6969), 10% Fetal Bovine Serum (Corning, 35-079-CV), L-Glutamine (2 mM; Thermo Fisher Scientific, 25030), penicillin/streptomycin (Thermo Fisher Scientific, 15140) and sodium pyruvate (Thermo Fisher Scientific, 11360-039). Cells were incubated at 37°C in a humidified atmosphere containing 5% CO_2_.

For assessment of cell cycle phase distribution, 10^6^ MM cells were cultured in RPMI supplemented with different arginine concentrations, washed twice with PBS after 24 hours, and fixed with 1 ml of cold ethanol 75% added drop by drop on vortex and stored overnight at -20°C. Pelleted cells were then washed twice with PBS and stained with 100 µl of PI solution [PBS; 2% IGEPAL CA-630 (Sigma-Aldrich I-3021); 0.2 mg/ml propidium iodide (Biolegend, 421301); 2 mg/ml PureLink™ RNase A (Invitrogen, 12091)] for 30 minutes at 37°C. After two washes in PBS, cells cycle data were acquired using a CytoFLEX Flow Cytometer (Beckman Coulter) and analysed with FCS Express software.

For assessment of bortezomib-induced toxicity (Cell Signaling Technology, 2204), cells were pre-treated with the selective AKT inhibitor MK2206 (Selleckchem, S1078) or left untreated and then stained using FITC Annexin V Apoptosis Detection Kit I (BD Pharmingen, 556547) following manufacturer’s instructions. Early apoptotic and dead cells were identified using a CytoFLEX Flow Cytometer (Beckman Coulter) and analysed with FCS Express software.

### Immunoblot analyses

Protein lysates were obtained from cells or excised tumors from transplanted mice through solubilization in lysis buffer [PBS; 1% Sodium Dodecyl Sulfate (SDS; Sigma-Aldrich 05030); PhosSTOP™ (Roche 04 906 845 001); cOmplete™ Protease Inhibitor Cocktail (Roche 11873580001)], separated on 8–15% SDS-PAGE in reducing conditions and transferred onto nitrocellulose membrane (GE Healthcare 10600002). Western blot (WB) analyses were performed following standard protocols. The following Abs were used: rat ant-IRF4 (1:500; Biolegend, 646402); rabbit anti-BLIMP1 (1:1000; Cell Signaling, 9115); rabbit anti-phospho eIF2α (1:1000; Cell Signaling, 3398); rabbit anti-eIF2α tot (1:1000; Cell Signaling, 5324); rabbit anti-phospho S6 Ribosomal Protein (1000; Cell Signaling, 5364); rabbit anti-S6 Ribosomal Protein (1000; Cell Signaling, 2317); rabbit anti-LC3B polyclonal Ab (1:500; Novus Biologicals, NB100–220); rabbit anti-phospho AKT (S473) (1:1000; Cell Signaling, 9271); rabbit anti-phospho AKT (T308) (1:1000; Cell Signaling, 2965) rabbit anti-Akt (pan) (C67E7) (1:1000; Cell Signaling, 4691); rabbit anti-phospho BAD (1:1000; Cell Signaling, 4366); rabbit anti-BAD tot (1:1000; Cell Signaling, 9239); mouse anti-ACTB/b actin (1:5000; Sigma-Aldrich, A5441). Quantifications were obtained with the gel analysis option of ImageJ software.

### Retrotranscription and real time qRT-PCR

Total RNA was extracted from cells or excised tumors from transplanted mice by homogenization in Trifast™ kit (EuroClone EMR507). RNA was reverse-transcribed using ImProm-II Reverse Transcription System (Promega, A3800) following manufacturer’s instructions. Expression of target genes was analyzed by qRT-PCR on LightCycler 480 (Roche) using LightCycler^®^ 480 SYBR Green I (Roche, RO04887352001). Specific primers were designed by using Primer 3 (primer sequences and PCR conditions available upon request). Amplification efficiency was determined by dilution curves, and expression of target transcripts normalized to H3. The relative mRNA expression of genes of interest was calculated using the Advanced Relative Quantification software (Roche).

### Animal study

Male and female young adult (4-month-old) Rag2^–/–^γc^–/–^ mice (kindly provided by Paolo Ghia, San Raffaele Scientific Institute) were subcutaneously implanted with 2*10^6^ H929 MM cells in the lateral lumbar region. When tumors were palpable, mice were divided in two equally sized sex-matched groups and treated with the clinical-grade arginase inhibitor CB1158, kindly supplied by Calithera Biosciences, Inc. and Incyte Corporation (100 µg/g of body weight), or vehicle, by oral gavage *bis in die*. Tumor growth was monitored daily by caliper assessment from the beginning of treatment, and mice sacrificed when tumors were in the imminence of reaching the critical volume of 4,000 mm^3^ (~18 days). Before sacrifice, blood samples were withdrawn by penetrating the retro-orbital sinus in mice with a sterile hematocrit capillary tube. Sera were obtained by centrifugation for 15 minutes at 3,000 rpm at room temperature. Arginine serum concentration was assessed by using competitive ELISA analysis (MyBioSource MBS726317), following the manufacture’s protocol. After sacrifice, tumors were explanted from the flanks of recipient mice and measured using calipers. Two small biopsies were obtained from each explanted tumors and used for extraction of proteins and RNA. The remaining tumor tissues were formalin-fixed and paraffin-embedded for histological examination. Hematoxylin and eosin staining was performed on 5 µm-thick tumor slices following standard protocols. For histological analysis, sections were viewed under a Zeiss Axioscope 40FL microscope (Carl Zeiss, Oberkochen, Germany), equipped with an AxioCam MRc5 digital video camera. Quantification of innate immune cell infiltration and of cellular death was performed on stained tumor sections by an observer unaware of the treatment. All animal procedures were carried out at San Raffaele Scientific Institute, Milan, Italy, according to, and approved by, the San Raffaele Institutional Animal Care and Use Committee.

### Statistical analyses

We performed comparisons between groups by using two-tailed unpaired or paired Student’s *t*-test or two-way ANOVA test followed by Bonferroni’s *post hoc* test, as indicated. Only statistically significant pairwise comparisons are indicated in the figures, with significance threshold set at p<0.05.

## Results

### Differential sensitivity of T lymphocytes and myeloma cells to low arginine

**Figure 1:**
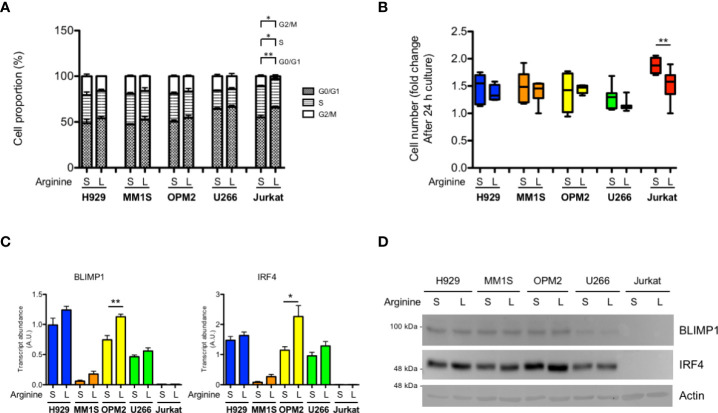
Differential sensitivity of T lymphocytes and myeloma cells to arginine depletion. MM cell lines (H929, MM1S, OPM2, U266) and immortalized human T lymphocytes (Jurkat) were cultured for 24 hours (h) in complete medium containing standard (S, 1140 µM) or low (L, 11 µM) arginine concentration. **(A)** Cell cycle distribution was analyzed by flow cytometry and the percentage of cells in the indicated phases are represented (n = 3 independent experiments). *p<0.05, **p<0.01, paired *t*-test. **(B)** Box and whiskers plot representing relative cell proliferation of the indicated cell lines in different arginine concentration for 24 h (n ≥ 6 experiments). **p<0.01, paired *t*-test. **(C)** Relative abundance of transcripts encoding BLIMP1 and IRF4 (A.U., arbitrary units; n ≥ 3 independent experiments). Bars indicate average ± s.e.m. *p<0.05, **p<0.01, unpaired t-test. **(D)** Representative immunoblot analysis showing the effect of 24 h arginine shortage on the protein abundance of the PC master factors BLIMP1 and IRF4 in MM cell lines. Actin B serves as loading control.

Arginine is a conditionally essential amino acid, as its synthesis relies on expression of the limiting enzymes, ASS1 and ASL within the urea cycle (16). Quantification by real time RT-qPCR analysis of *ASS1* and *ASL* transcripts showed increased expression when MM cell lines were cultured in medium containing low arginine concentration (11 µM), as compared to cells cultured in standard arginine concentration (1140 µM) (**Supplementary Figure 1**), indicating that MM cells respond to arginine deprivation by increasing its biosynthesis. Since T cells rely on exogenous arginine supply (9, 10), we wondered whether myeloma cells are less sensitive to arginine deprivation than T cells. To test this hypothesis, we cultured a panel of human MM cell lines, representative of different genetic landscapes in human disease, and a representative human immortalized T cell line (Jurkat) in medium containing standard or low arginine concentration. Reduced arginine had no effect on cell survival in MM and T cells (not shown); however, it induced significant accumulation of T cells in S and G_0_/G_1_ phases, at the expense of G_2_/M, but failed to alter cell cycle distribution of myeloma cell lines (**Figure 1A**). Cell cycle effects were reflected by a differential impact on cell proliferation, as low arginine significantly reduced T cell growth in as little as 24 hours, while sparing proliferation of MM cell lines (**Figure 1B**). If exposed for longer time to low arginine, MM cells kept growing, but exhibited modest and inconsistently significant decreases in proliferation (not shown). These results indicate that MM cells are less vulnerable to arginine deprivation than T cells. Moreover, analysis of the expression of the PC master transcriptional regulators, BLIMP1 and IRF4, showed no reduction in MM cells cultured in low arginine media, at neither transcript (**Figure 1C**) nor protein (**Figure 1D**) level. In fact, low arginine induced invariably higher expression of both transcripts, which reached significance in OPM2 cells (**Figure 1C**). Taken together, these results prompted us to test if arginine depletion may also exert direct beneficial effects on MM cells.

### Arginine shortage does not activate the integrated stress response but reduces mTORC1 activity

**Figure 2:**
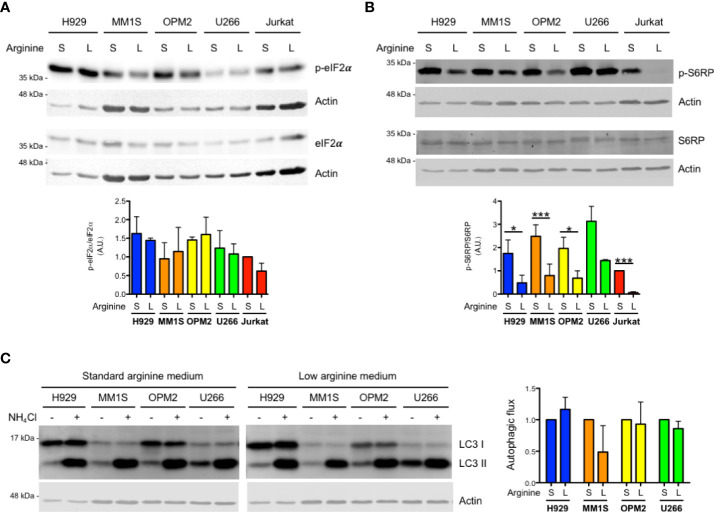
Arginine shortage does not activate the integrated stress response but inhibits mTORC1 in myeloma cells. **(A, B)** Representative immunoblot analyses showing expression of total and phosphorylated eIF2α **(A)** or total and phosphorylated S6RP **(B)** in MM (H929, MM1S, OPM2, U266) and Jurkat cells after 24 h of culture in complete medium containing standard (S) or low (L) arginine concentration as in **Figure 1**. Actin B serves as loading control. **(A, B)** Bottom histograms show quantification of phosphorylated relative to total protein pools (n = 3 independent experiments; A.U., arbitrary unit). Bars indicate average ± s.d. *p<0.05, ***p<0.001, unpaired *t*-test. **(C)** Left: representative immunoblot analysis of endogenous unconjugated LC3-I and lipid-conjugated LC3-II in MM lines after 24 h of culture in different arginine concentrations. Cells were treated for 2 h with 50 mM NH_4_Cl or left untreated prior to 1% SDS lysis. Actin B serves as loading control. Right: histogram representing quantification of autophagic flux, estimated as the difference of abundance between LC3II after 2 h NH_4_Cl treatment and LC3II at steady state in complete medium containing standard (S) or low (L) arginine concentration (n = 3 independent experiments). Bars indicate average ± s.d.

We then looked for stress-adaptive cellular pathways that could be modulated by arginine deprivation in MM cells. Complete arginine starvation is known to induce activation of the ISR by stimulating stress kinases, which in turn may attenuate general protein translation *via* phosphorylation of eIF2α. These include GCN2 (general control nonderepressible 2), capable of sensing arginine depletion *via* direct engagement by uncharged tRNAs, and PERK (protein kinase R-like endoplasmic reticulum kinase), *via* induction of ER stress due to protein misfolding. However, immunoblot analyses of the expression of phosphorylated and total eIF2α showed no induction of eIF2α phosphorylation, indicative of no ISR activation, in MM lines in response to arginine deprivation (**Figure 2A**).

Intracellular reduction of arginine can also be sensed by CASTOR1, which in turn may reduce mTORC1 activity, with potentially myeloma-relevant downstream effects, including increased autophagy or pro-survival effects *via* positive regulation of mTORC2 complex activity. Quantification of immunoblot analysis of phosphorylated ribosomal protein S6 (S6RP), an established downstream target and effector of mTORC1, showed significant mTORC1 inhibition by arginine deprivation in MM cell lines (**Figure 2B**). Notably, in the same culture conditions, S6RP phosphorylation was abolished in Jurkat T cells (**Figure 2B**), again attesting to higher sensitivity to arginine starvation of T lymphocytes. Moreover, S6RP phosphorylation was dose-dependently modulated by arginine abundance in MM cells, where it was abolished in the complete absence of arginine, recapitulating the profound inhibition achieved by the paradigmatic mTORC1 inhibitor rapamycin (**Supplementary Figure 2**). To challenge the functional relevance of the observed partial inhibition of mTORC1 by arginine shortage, we first tested if it may increase autophagy. To this end, MM cells cultured in standard or low arginine were treated with ammonium chloride to block lysosomal activity and the rate of lysosomal digestion of lipidated LC3 (LC3-II) assessed as a measure of total autophagic flux. This experiment revealed that the partial inhibition of mTORC1 induced by arginine deprivation is not sufficient to increase autophagy in MM cells (**Figure 2C**).

### Low arginine induces AKT phosphorylation and activity in selected MM cell lines

**Figure 3:**
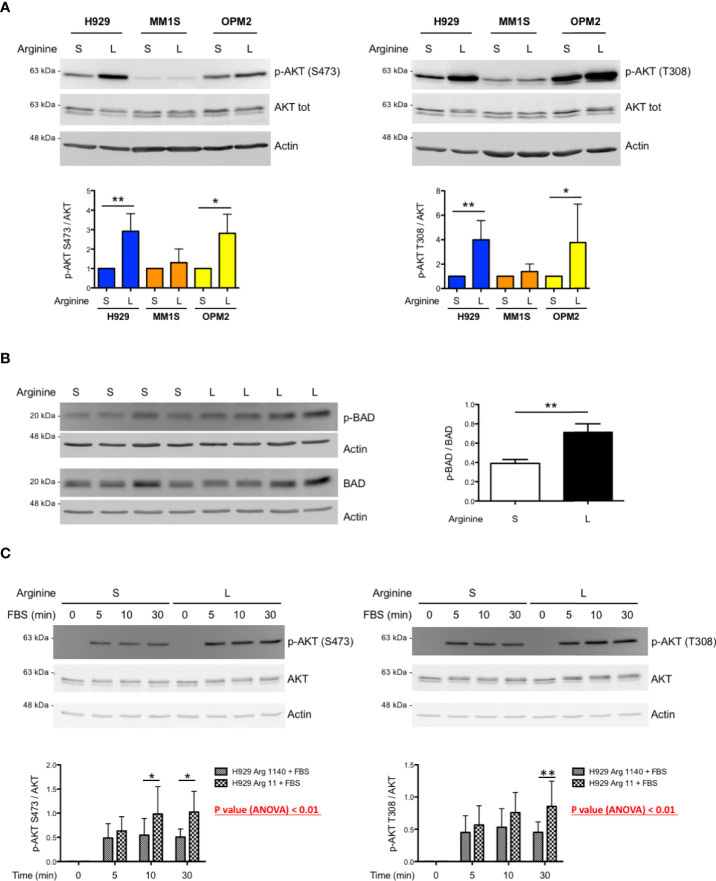
Low arginine induces AKT phosphorylation and activity in selected MM cell lines. **(A)** Representative immunoblot analyses showing expression of total and phosphorylated AKT at serine 473 (left upper panel) and at threonine 308 (right upper panel) in the indicated MM cell lines after 24 h of culture in complete medium containing standard (S) or low (L) arginine concentration (actin B serving as loading control). Histograms (lower panels) show quantification of phosphorylated AKT relative to total protein (n ≥ 5 independent experiments). Bars indicate average ± s.d. *p<0.05, **p<0.01, paired *t*-test. **(B)** Representative immunoblot analysis showing expression of total and phosphorylated BAD at serine 99 (left panel) in H929 cells after 24 h of culture in complete medium containing standard (S) or low (L) arginine concentration (actin B serves as loading control). Histogram (right panel) shows quantification of the phosphorylated relative to total BAD (n = 4 independent experiments). Bars indicate average ± s.d. **p<0.01, paired *t*-test. **(C)** Representative immunoblot analyses showing expression of total and phosphorylated AKT at serine 473 (left upper panel) and at threonine 308 (right upper panel) in H929 cells after FBS stimulation. Following 16 h incubation in medium without FBS and containing standard (S) or low (L) arginine concentrations, as above, cells were stimulated with FBS (10% v/v) for the indicated time. Actin B serves as loading control. Histograms (lower panels) show quantification of the phosphorylated relative to total AKT (n = 5 independent experiments) after FBS stimulation for the indicated time. Bars indicate average ± s.d. *p<0.05, **p<0.01, Bonferroni posttests after 2-way ANOVA.

The mTORC1 complex can control mTORC2 activity *via* S6 kinase 1 (S6K1) that, in turn, inhibits phosphoinositide 3-kinase (PI3K) signalling (20). Therefore, we asked whether arginine deprivation in myeloma cells could induce mTORC2, possibly resulting in increased AKT phosphorylation and activity. In keeping with this hypothesis, immunoblot analyses showed increased AKT phosphorylation in total lysates from most MM cell lines cultured in low arginine medium for 24 hours at both threonine 308 and serine 473 residues (**Figure 3A**), two modifications known to determine full AKT activation. Increased AKT phosphorylation at serine 473 was confirmed in additional MM lines (**Supplementary Figure 3**). To further confirm the positive effect of low arginine on AKT activity, we analysed the phosphorylation status of the BH3-only protein BAD, an established direct AKT target whose phosphorylation at serine 99 or 136, respectively in the human and murine counterpart, blocks its pro-apoptotic function (21). As shown in **Figure 3B**, MM cells cultured in low arginine showed a significant increase in BAD phosphorylation at serine 99. We then sought to test if the effect of arginine shortage on AKT phosphorylation depends on PI3K, typically activated by receptor tyrosine kinases (RTKs). When MM cells were cultured in standard or low arginine medium in the absence of foetal bovine serum (FBS) we observed an almost complete disappearance of AKT phosphorylation (**Figure 3C**). Moreover, canonical stimulation of RTKs with either FBS or fibroblast growth factor 2 (FGF2) induced rapid increases in AKT phosphorylation at both threonine 308 and serine 473, that were significantly higher when cells were cultured in low arginine medium (**Figure 3C** and **Supplementary Figure 4**). Taken together, the data reveal a direct stimulatory effect of low arginine on PI3K-dependent AKT activity, possibly exerting a pro-survival role in MM cells.

### Partial arginine deprivation reduces bortezomib-induced cell death in MM cell lines by increasing AKT phosphorylation

**Figure 4:**
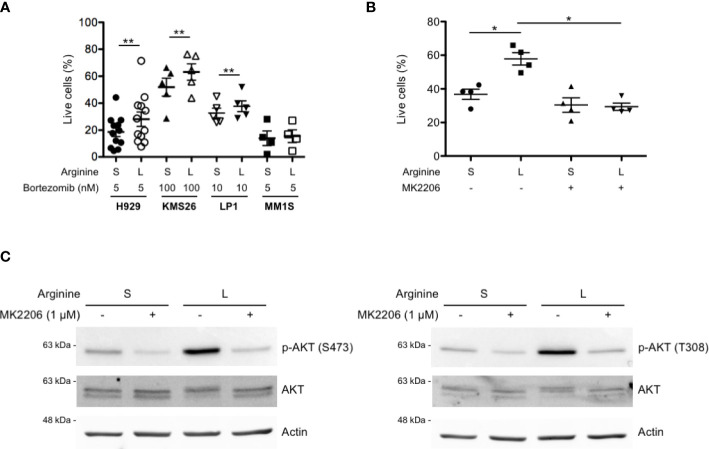
Partial arginine deprivation reduces bortezomib-induced cell death in MM cells by increasing AKT phosphorylation. **(A)** MM cell lines were cultured in complete medium containing standard (S) or low (L) arginine concentration, as in previous figures. After 8 h, cells were treated with the indicated doses of bortezomib for 16 h and stained with fluorescent Annexin V and propidium iodide. Dead and apoptosis-committed cells were identified by flow cytometry and percentages of live cells represented as scatter plots (mean values, n ≥ 4 independent experiments). **p<0.01, paired t-test. **(B)** H929 cells were cultured in different arginine concentrations, as above, and treated with the AKT inhibitor MK2206 (1 µM) or left untreated. After 8 h, cells were treated with bortezomib (2.5 nM) for 16 h and stained with fluorescent Annexin V and propidium iodide. Dead and apoptosis-committed cells were counted by flow cytometry and live cell percentages represented as scatter plots (mean values, n = 4 independent experiments). *p<0.05, paired t-test. **(C)** Representative immunoblot analyses showing expression of total and phosphorylated AKT at serine 473 (left panel) and at threonine 308 (right panel) after 24 h of culture in S or L arginine concentrations in H929 cells treated with MK2206 (1 µM) or left untreated. Actin B serves as loading control.

We then tested whether increased AKT phosphorylation by partial arginine starvation could protect MM cells from cell death. To this end, a panel of MM lines were cultured in standard or low arginine and treated with the first-in-class proteasome inhibitor bortezomib (Btz) at different doses, to achieve substantial cell death, based on each line’s carefully determined apoptotic sensitivity (not shown). We found a significant reduction of Btz-induced cell death in most cell lines cultured in low arginine medium (**Figure 4A**). To test if the observed death-preventing effect is mediated by increased activity of the PI3K/AKT pathway, we used the allosteric AKT inhibitor MK2206 (33, 34). Pre-treatment with MK2206 significantly prevented the protective effect of arginine deprivation on Btz-induced cell death in MM cells (**Figure 4B**) while reducing AKT phosphorylation (**Figure 4C**). Taken together, the data show that arginine deprivation may exert a pro-survival effect on myeloma cells *via* increased AKT phosphorylation.

### Arginase inhibition reduces myeloma growth in immunocompromised recipient mice

**Figure 5:**
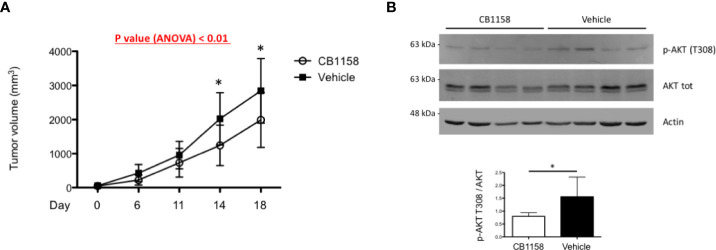
Pharmacological arginase inhibition reduces myeloma growth in immunocompromised recipient mice. **(A)** 2x10^6^ H929 cells were implanted subcutaneously in the flank of Rag^–/–^γc^–/–^ mice. When tumors were palpable, mice were randomly assigned to two groups and treated with the arginase inhibitor CB1158 (100 µg/g body weight) or vehicle by oral gavage *bis in die* for 18 days. Growth of subcutaneous tumors was measured by caliper and represented as mean size (mm^3^) ± s.d. (n = 7 mice/group). *p<0.05, Bonferroni posttests after 2-way ANOVA. **(B)** Representative immunoblot analyses of protein extracts from individual tumor biopsies at the end of the study. Expression of total and phosphorylated AKT at threonine 308 (upper panel) are shown (actin B serving as loading control). Histogram (lower panel) shows quantification of phospho-ATK relative to total protein (n = 7 mice/group). Bars indicate average ± s.d. *p<0.05, unpaired *t*-test.

Having identified a protective pro-survival role of arginine deprivation *in vitro*, we then sought to challenge its relevance *in vivo*. To gauge the potential benefit of raising arginine against myeloma growth independently of expected effects on adaptive immunity, we xenotransplanted human myeloma cells subcutaneously into young adult T cell-deficient Rag2^–/–^γc^–/–^ recipient mice. When tumors became palpable, mice were treated with the clinical-grade arginase inhibitor CB1158 or vehicle enterally every 12 hours, and tumor growth monitored by caliper assessment. Mice were euthanized just before tumors reached the critical volume of 4,000 mm^3^. Arginase inhibition induced significantly higher serum arginine concentrations in Rag2^–/–^γc^–/–^ mice (**Supplementary Figure 5A**). Despite treatment started when tumors were rather large, their growth was significantly reduced by arginase inhibition (**Figure 5A**). Histopathological (not shown) and expression analyses of CD45 and CD68 (**Supplementary Figure 5B**) confirmed tumor volume differences to be accounted for by variations in PC amounts, and not by differential accumulation of microenvironmental innate immune cells. Moreover, immunoblot analyses revealed lower AKT phosphorylation in lysates of tumor biopsies obtained from CB1158-treated as compared to vehicle-treated recipients (**Figure 5B**). Collectively, our findings confirm a positive, T cell-independent role of low arginine on MM growth *in vivo.*

## Discussion

From a genetic perspective, MM is a very heterogeneous disease. Remarkable efforts in the last decade have characterized the mutational profiles and the genetic bases of clonal evolution of myeloma (35, 36) providing critical insight on the impact of therapeutic pressure on its genetic architecture, conducive to chemoresistance and disease relapse (37). However, despite the growing knowledge about genetic abnormalities in patients, with relevant implications for personalized treatments, myeloma remains incurable. Targeting cancer-specific non-oncogene addictions offers additional valuable therapeutic avenues (38). MM is the paradigmatic cancer responsive to proteasome inhibitors (PI), in view of its reliance on cellular protein homeostatic pathways for survival (39, 40). Cancers also depend on immunosuppressive circuits, which could also be targeted therapeutically (38). Indeed, the clinical introduction of PIs and immunomodulatory agents led to a substantial improvement of the survival of myeloma patients (41).

It is currently accepted that MM progression depends on a complex, multi-directional cross-talk between cancer cells and the BM multicellular microenvironment (42), which includes diverse types of immune cells. A number of mechanisms of cancer immunoevasion have been characterized, whose therapeutic manipulation may improve patient survival (43). Exemplary are approaches targeting immune checkpoints by interfering with lymphocyte inhibitory molecules (*e.g.*, CTLA-4, PD-1 or PD-L1) that may induce long-term therapeutic effects (43, 44). Recently, interest grew in MDSCs, a heterogeneous class of myeloid cells that populate the tumor microenvironment and exert immunosuppressive activity through a variety of mechanisms (6). Little is known on the physiologic role of MDSCs on B lymphocyte ontogeny, apart from recent reports proposing direct roles reducing proliferation of human mature B cells and inhibiting antibody production (45, 46). However, MDSCs have been associated with MM pathobiology. Although they have not been implicated in malignant transformation of B cells, MM cells were shown to induce MDSC expansion (2, 3, 47), which, in turn, may promote myeloma growth (5, 48). Among diverse immunoevasive and pro-tumoral strategies, several studies have identified the expression of arginase in MDSCs as a key mechanism mediating suppression of anti-tumor T cell responses, both in patients and animal cancer models (44) and validated pharmacological arginase inhibition as an effective therapeutic strategy in cancer mouse models (49). As a result, orally available compounds targeting arginase have recently been and are currently being evaluated in clinical trials in solid tumors and in MM (44).

Arginine is a conditionally essential amino acid whose shortage is expected to exert profound metabolic effects in cells, mainly *via* inhibition of mTORC1 and induction of the ISR *via* the amino acid starvation-sensing kinase GCN2 (17, 18). In this study, we reasoned that both effects may be beneficial to MM cells, *e.g.*, by curtailing their constitutive proteosynthetic stress or by inducing stress-adaptive and pro-survival strategies, including autophagy. Following this hypothesis, we hereby adopted a reductionist approach to formally explore the possibility of direct beneficial effects of arginine depletion on MM cells, not only to challenge the rational adoption of arginase inhibition, but also as a framework to investigate addictions of therapeutic significance.

In this work, we first documented higher vulnerability of the proliferation of T cells to low arginine than that observed in MM cell lines; next, we exploited an arginine concentration that revealed such differential sensitivity as an experimental window to unveil possible direct beneficial effects of low arginine on MM cells. At low arginine concentration, MM cells failed to upregulate the ISR, as demonstrated by unmodified eIF2α phosphorylation levels, implying that GCN2 was not activated. This could be explained by sufficient arginine availability, possibly sustained by the high intracellular protein turnover characteristic of PCs. However, in this experimental setting arginine depletion resulted in sizeable inhibition of mTORC1 activity, which did not raise overall autophagic flux, a finding in apparent contradiction with the established negative effect of mTORC1 on autophagy (19). Indeed, the mTORC1 inhibitor rapamycin is universally used to stimulate autophagy across different cell types, including MM cells (27). However, standard autophagy-inducing rapamycin treatment resulted in more profound inhibition of mTORC1 than that achieved by low arginine. Indeed, we were able to induce comparable mTORC1 inhibition only by completely depleting arginine from the culture media. This observation suggests the existence of a poorly characterized threshold of mTORC1-dependent regulation of autophagy, whose details and functional relevance may deserve future investigations.

Our study offers original *in vitro* evidence that arginine depletion may stimulate the PI3K/AKT pathway in MM cells and protect from cell death, as it reduced the pro-apoptotic impact of the first-in-class PI bortezomib, an effect possibly mediated, at least in part, by the inhibitory phosphorylation of the BH3-only protein BAD. By heterodimerizing with Bcl-2 or Bcl-XL, active BAD disrupts their inhibitory association with the mitochondrial outer membrane permeabilizing effectors Bax and Bak (50). AKT-driven BAD phosphorylation inhibits its pro-apoptotic function (51). PI3K signaling is activated by several cytokines and factors that sustain myeloma growth and plays a major role in controlling MM cell proliferation and apoptosis and is thus becoming attractive as a therapeutic target against myeloma (30). However, therapeutic attempts to interfere with the mTOR/PI3K/AKT pathway using mTORC1 inhibitors (*e.g.*, rapalogs) demonstrated low efficacy against myeloma because of induction of negative feedback circuits increasing mTORC2 activity (30, 31). Our findings encourage the adoption of compounds targeting directly PI3K or AKT activity in preclinical and clinical studies, also in combination with other pharmacological treatments (31).

In our *in vitro* experiments, arginine depletion induced higher AKT phosphorylation in all human MM lines tested, except for MM1S cells. This finding prompted us to investigate differential features possibly underlying this biological response. A notable discriminant was the t(4;14) translocation involving the immunoglobulin heavy chain (IGH) region. Found in about 11% MM patients (1), this translocation is associated with poor prognosis, with no improvement in patient survival observed in the past two decades (52), urging to investigate related pathobiology and vulnerabilities in this group. In our study, harboring t(4;14) invariably coincided with displaying higher AKT phosphorylation in response to arginine deprivation. This translocation is associated with higher expression of the myeloma SET domain protein (MMSET) and of the fibroblast growth factor receptor 3 (FGFR3). By placing the gene encoding FGFR3, a transmembrane tyrosine kinase receptor, under control of the active IGH promoter, t(4;14) may enhance ligand-dependent and -independent signaling. Aberrant FGFR3 signaling may lead to activation of downstream pathways that drive disease progression, including the mTOR/PI3K/AKT axis. Our data raise the testable hypothesis that overexpression of FGFR3 may be responsible for the increased AKT phosphorylation and pro-survival effect observed in response to partial arginine deprivation.

Arginine depletion in the tumor microenvironment is acknowledged as a key mechanism mediating suppression of anti-tumor immunity, and restoration of normal environmental arginine concentration has been proposed as a promising immune-modulatory strategy against cancers, including myeloma. Our findings reveal an unprecedented direct mechanism whereby low arginine may favor myeloma growth, in addition to its already known immunosuppressive activity. To test the relevance of direct arginine-mediated PC autonomous effects on myeloma growth *in vivo*, we employed the arginase inhibitor, CB1158 to restore arginine concentration in myeloma xenografts into Rag2^–/–^γc^–/–^ immunocompromised recipient mice. In this model, lacking the expected therapeutic advantage from restored T cell immunity, an impact on tumor growth, albeit modest, should be accounted for by T-independent direct effects on PCs. Moreover, although earlier arginase inhibition would have inhibited tumor growth more profoundly, CB1158 administration was initiated only when tumors were already palpable, to rigorously control for heterogeneous growth rates. Despite these very stringent conditions, the study revealed a significant impact of arginine abundance on tumor growth, which provides proof-of-principle evidence of the pathophysiological relevance of arginine concentration to the intrinsic biology of MM cells. The *in vivo* association of lower AKT phosphorylation with reduced tumor growth upon arginase inhibition is consistent with increased adaptation to environmental stress and survival of MM cells in the presence of reduced arginine availability. Together with our *in vitro* observations, these findings also suggest the possible therapeutic advantage of combining pharmacological arginase and proteasome inhibition.

In conclusion, in the present study we identified a direct beneficial effect of arginine depletion on MM cells, which may play a role in disease progression. In particular, in reductionist *in vitro* studies, we found that low arginine may protect MM cells from drug-induced cell death, an effect accounted for by increased mTORC2-driven AKT activity, and possibly, at least in part, by heightened BAD phosphorylation. We then challenged the potential significance of this axis *in vivo* by pharmacological arginase inhibition in a xenograft mouse myeloma model. We speculate that our proof-of-principle evidence may contribute to reveal previously unidentified vulnerabilities of therapeutic value.

## Data availability statement

The original contributions presented in the study are included in the article/**Supplementary Material**. Further inquiries can be directed to the corresponding author.

## Ethics statement

The animal study was reviewed and approved by the Institutional Animal Care and Use Committee, Ospedale San Raffaele, Milano, Italy.

## Author contributions

Conceptualisation and study design, MT and SC. Experimental design, experimental execution, and data analyses, MT, LO, UO, FS, MP, and SC. Critical discussion and interpretation, MT, LO, and SC. Writing, MT and SC. Critical reading, MT, LO, UO, AR, FDR, MP, and SC. Funding acquisition and supervision, SC. All authors contributed to the article and approved the submitted version.

## Funding

This work was supported by research grants from Fondazione AIRC (Investigator Grant 2016 - 18858 and IG 2019 - 23245), International Myeloma Society (IMS) and Paula and Rodger Riney Foundation (Translational Research Award 2021 and 2022), and Fondazione Cariplo (2018-0541) to SC and from the International Myeloma Foundation (2017 Brian D. Novis Junior Research Award) to AR.

## Acknowledgments

We are thankful to Federica Loro and Roberta Colzani for administrative assistance and to all members of the Cenci lab for creative discussions and scientific advice. We thank Giovanni Tonon for sharing MM lines and for critical advice; Paolo Ghia and Jessica Bordini for critical support with mouse studies; Alessandra Boletta and Gianfranco Distefano for scientific discussions and reagents; Niccolò Bolli for supervision and scientific advice. We are thankful to Calithera Biosciences, Inc. and Incyte Corporation for the supply of the arginase inhibitor CB1158.

## Conflict of interest

The authors declare that the research was conducted in the absence of any commercial or financial relationships that could be construed as a potential conflict of interest.

## Publisher’s note

All claims expressed in this article are solely those of the authors and do not necessarily represent those of their affiliated organizations, or those of the publisher, the editors and the reviewers. Any product that may be evaluated in this article, or claim that may be made by its manufacturer, is not guaranteed or endorsed by the publisher.

## Supplementary material

Supplementary Figure 1 Arginine shortage induces expression of urea cycle limiting enzymes. The indicated multiple myeloma (MM) cell lines were cultured for 24 hours (h) in complete medium containing standard (S) or low (L) arginine concentration (1140 µM or 11 µM, respectively). The histogram shows relative abundance of transcripts encoding ASS1 and ASL (real-time qRT-PCR; A.U., arbitrary unit). Bars indicate average of 4 independent experiments ± s.e.m. *p<0.05; ***p<0.001, unpaired *t*-test.

Supplementary Figure 2 mTORC1 is partially inhibited in cells cultured in low arginine. **(A)** Representative immunoblot analysis showing expression of total and phosphorylated S6RP in H929 MM cells after 24 h of culture in complete medium containing the indicated arginine concentrations (actin B serves as loading control; n≥3 independent experiments). **(B)** Immunoblot analysis showing the effect of 24 h treatment with the indicated doses of the established mTORC1 inhibitor, rapamycin on S6RP phosphorylation in H929 MM cells (n ≥ 3 independent experiments).

Supplementary Figure 3 Low arginine induces AKT phosphorylation in MM cell lines. Representative immunoblot analysis from ≥3 independent experiments showing expression of total and phosphorylated AKT at serine 473 (S473) in two independent experiments in KMS26 (left panel) or LP1 (right panel) MM cell lines after 24 h of culture in complete medium containing standard (S) or low (L) arginine concentration (1140 µM or 11 µM, respectively). Actin B serves as loading control.

Supplementary Figure 4 Arginine shortage enhances FGF2-induced AKT phosphorylation. Representative immunoblot analysis from ≥3 independent experiments showing expression of total and phosphorylated AKT at serine 473 (S473) (left upper panel) and total and phosphorylated AKT at threonine 308 (T308) (right upper panel) in H929 cell line after FGF2 stimulation. Cells were cultured in medium without FBS and containing standard (S) or low (L) arginine concentration and, after 16 h, stimulated with FGF2 (10 ng/ml) for the indicated time (actin B serves as loading control). Histograms (lower panels) show quantification of phosphorylated relative to total AKT (n = 3 independent experiments) after FGF2 stimulation for the indicated time. Bars indicate average ± s.d. 2-way ANOVA.

Supplementary Figure 5 Effect of pharmacological arginase inhibition on circulating L-arginine concentration and intratumoral expression of myelomonocytic markers in myeloma-bearing Rag^–/–^γc^–/–^ mice. **(A)** Serum arginine concentration in tumor-bearing Rag^–/–^γc^–/–^ mice treated with CB1158 or vehicle. Box and whiskers plot showing L-arginine concentration in sera from Rag^–/–^γc^–/–^ mice xenotransplanted subcutaneously with H929 MM cells and treated with the arginase inhibitor CB1158 (100 µg/g of body weight) or vehicle by oral gavage *bis in die* for 18 days. L-arginine concentration was quantified by ELISA at the end of treatment (n = 7 mice/group). **p<0.01, unpaired *t*-test. **(B)** Relative abundance of transcripts encoding CD45 (common leukocyte marker) and CD68 (macrophage marker) in tumor biopsies at sacrifice (A.U., arbitrary units; n = 7 mice/group; average ± s.e.m.).

## References

[1] Kumar SK Rajkumar V Kyle RA van Duin M Sonneveld P Mateos M-V . Multiple myeloma. Nat Rev Dis Primer (2017) 3:1–20. doi: 10.1038/nrdp.2017.46 28726797

[2] De Veirman K Van Valckenborgh E Lahmar Q Geeraerts X De Bruyne E Menu E . Myeloid-derived suppressor cells as therapeutic target in hematological malignancies. Front Oncol (2014) 4:349. doi: 10.3389/fonc.2014.00349 25538893 PMC4258607

[3] De Veirman K Van Ginderachter JA Lub S De Beule N Thielemans K Bautmans I . Multiple myeloma induces mcl-1 expression and survival of myeloid-derived suppressor cells. Oncotarget (2015) 6:10532–47. doi: 10.18632/oncotarget.3300 PMC4496373 25871384

[4] Giallongo C Tibullo D Parrinello NL Cava PL Rosa MD Bramanti V . Granulocyte-like myeloid derived suppressor cells (G-MDSC) are increased in multiple myeloma and are driven by dysfunctional mesenchymal stem cells (MSC). Oncotarget (2016) 7:85764–75. doi: 10.18632/oncotarget.7969 PMC5349872 26967390

[5] Perez C Botta C Zabaleta A Puig N Cedena M-T Goicoechea I . Immunogenomic identification and characterization of granulocytic myeloid-derived suppressor cells in multiple myeloma. Blood (2020) 136:199–209. doi: 10.1182/blood.2019004537 32325491

[6] Gabrilovich DI . Myeloid-derived suppressor cells. Cancer Immunol Res (2017) 5:3–8. doi: 10.1158/2326-6066.CIR-16-0297 28052991 PMC5426480

[7] Lee SJ Borrello I . Role of the immune response in disease progression and therapy in multiple myeloma. In: Roccaro AM Ghobrial IM , editors. Plasma Cell Dyscrasias Cancer Treatment and Research. Cham, Switzerland: Springer International Publishing (2016). p. 207–25. doi: 10.1007/978-3-319-40320-5_12 27696265

[8] Bronte V Zanovello P . Regulation of immune responses by l-arginine metabolism. Nat Rev Immunol (2005) 5:641–54. doi: 10.1038/nri1668 16056256

[9] Rodriguez PC Quiceno DG Ochoa AC . L-arginine availability regulates T-lymphocyte cell-cycle progression. Blood (2007) 109:1568–73. doi: 10.1182/blood-2006-06-031856 PMC1794048 17023580

[10] Geiger R Rieckmann JC Wolf T Basso C Feng Y Fuhrer T . L-arginine modulates T cell metabolism and enhances survival and anti-tumor activity. Cell (2016) 167:829–842.e13. doi: 10.1016/j.cell.2016.09.031 27745970 PMC5075284

[11] Van Valckenborgh E Schouppe E Movahedi K De Bruyne E Menu E De Baetselier P . Multiple myeloma induces the immunosuppressive capacity of distinct myeloid-derived suppressor cell subpopulations in the bone marrow. Leukemia (2012) 26:2424–8. doi: 10.1038/leu.2012.113 22522789

[12] Ramachandran IR Martner A Pisklakova A Condamine T Chase T Vogl T . Myeloid-derived suppressor cells regulate growth of multiple myeloma by inhibiting T cells in bone marrow. J Immunol (2013) 190:3815–23. doi: 10.4049/jimmunol.1203373 PMC3608837 23460744

[13] Lv M Wang K Huang X . Myeloid-derived suppressor cells in hematological malignancies: friends or foes. J Hematol Oncol.J Hematol Oncol (2019) 12:105. doi: 10.1186/s13045-019-0797-3 31640764 PMC6805310

[14] Lind DS . Arginine and cancer. J Nutr (2004) 134:2837S–41S. doi: 10.1093/jn/134.10.2837S 15465796

[15] Grohmann U Bronte V . Control of immune response by amino acid metabolism. Immunol Rev (2010) 236:243–64. doi: 10.1111/j.1600-065X.2010.00915.x 20636821

[16] Albaugh VL Pinzon-Guzman C Barbul A . Arginine metabolism and cancer. J Surg Oncol (2017) 115:273–80. doi: 10.1002/jso.24490 PMC6486789 27861915

[17] Pakos-Zebrucka K Koryga I Mnich K Ljujic M Samali A Gorman AM . The integrated stress response. EMBO Rep (2016) 17:1374–95. doi: 10.15252/embr.201642195 PMC5048378 27629041

[18] Chantranupong L Scaria SM Saxton RA Gygi MP Shen K Wyant GA . The CASTOR proteins are arginine sensors for the mTORC1 pathway. Cell (2016) 165:153–64. doi: 10.1016/j.cell.2016.02.035 PMC4808398 26972053

[19] Dibble CC Manning BD . Signal integration by mTORC1 coordinates nutrient input with biosynthetic output. Nat Cell Biol (2013) 15:555–64. doi: 10.1038/ncb2763 PMC3743096 23728461

[20] Julien L-A Carriere A Moreau J Roux PP . mTORC1-activated S6K1 phosphorylates rictor on threonine 1135 and regulates mTORC2 signaling. Mol Cell Biol (2010) 30:908–21. doi: 10.1128/MCB.00601-09 PMC2815569 19995915

[21] Hayakawa J Ohmichi M Kurachi H Kanda Y Hisamoto K Nishio Y . Inhibition of BAD phosphorylation either at serine 112 *via* extracellular signal-regulated protein kinase cascade or at serine 136 *via* akt cascade sensitizes human ovarian cancer cells to cisplatin. Cancer Res (2000) 60:5988–94. 11085518

[22] Cenci S van Anken E Sitia R . Proteostenosis and plasma cell pathophysiology. Curr Opin Cell Biol (2011) 23:216–22. doi: 10.1016/j.ceb.2010.11.004 21169004

[23] Meister S Schubert U Neubert K Herrmann K Burger R Gramatzki M . Extensive immunoglobulin production sensitizes myeloma cells for proteasome inhibition. Cancer Res (2007) 67:1783–92. doi: 10.1158/0008-5472.CAN-06-2258 17308121

[24] Bianchi G Oliva L Cascio P Pengo N Fontana F Cerruti F . The proteasome load versus capacity balance determines apoptotic sensitivity of multiple myeloma cells to proteasome inhibition. Blood (2009) 113:3040–9. doi: 10.1182/blood-2008-08-172734 19164601

[25] Fucci C Resnati M Riva E Perini T Ruggieri E Orfanelli U . The interaction of the tumor suppressor FAM46C with p62 and FNDC3 proteins integrates protein and secretory homeostasis. Cell Rep (2020) 32:108162. doi: 10.1016/j.celrep.2020.108162 32966780

[26] Pengo N Scolari M Oliva L Milan E Mainoldi F Raimondi A . Plasma cells require autophagy for sustainable immunoglobulin production. Nat Immunol (2013) 14:298–305. doi: 10.1038/ni.2524 23354484

[27] Milan E Perini T Resnati M Orfanelli U Oliva L Raimondi A . A plastic SQSTM1/p62-dependent autophagic reserve maintains proteostasis and determines proteasome inhibitor susceptibility in multiple myeloma cells. Autophagy (2015) 11:1161–78. doi: 10.1080/15548627.2015.1052928 PMC4590585 26043024

[28] Licari E Sánchez-del-Campo L Falletta P . The two faces of the integrated stress response in cancer progression and therapeutic strategies. Int J Biochem Cell Biol (2021) 139:106059. doi: 10.1016/j.biocel.2021.106059 34400318

[29] Tian X Zhang S Zhou L Seyhan AA Hernandez Borrero L Zhang Y . Targeting the integrated stress response in cancer therapy. Front Pharmacol (2021) 12. doi: 10.3389/fphar.2021.747837 PMC8498116 34630117

[30] Hu J Hu W-X . Targeting signaling pathways in multiple myeloma: Pathogenesis and implication for treatments. Cancer Lett (2018) 414:214–21. doi: 10.1016/j.canlet.2017.11.020 29174802

[31] Eichner R Fernández-Sáiz V Targosz B-S Bassermann F . Chapter six - cross talk networks of mammalian target of rapamycin signaling with the ubiquitin proteasome system and their clinical implications in multiple myeloma. In: Galluzzi L , editor. International review of cell and molecular biology. Cambridge, MA, USA: Academic Press (2019) p. 219–97. doi: 10.1016/bs.ircmb.2018.06.001 30712673

[32] Mimura N Hideshima T Anderson KC . Novel therapeutic strategies for multiple myeloma. Exp Hematol (2015) 43:732–41. doi: 10.1016/j.exphem.2015.04.010 PMC4540643 26118499

[33] Kumar Pal S Reckamp K Yu H Figlin RA . Akt inhibitors in clinical development for the treatment of cancer. Expert Opin Investig Drugs (2010) 19:1355–66. doi: 10.1517/13543784.2010.520701 PMC3244346 20846000

[34] Ramakrishnan V Kimlinger T Haug J Painuly U Wellik L Halling T . Anti-myeloma activity of akt inhibition is linked to the activation status of PI3K/Akt and MEK/ERK pathway. PloS One (2012) 7:e50005. doi: 10.1371/journal.pone.0050005 23185517 PMC3503708

[35] Bolli N Avet-Loiseau H Wedge DC Van Loo P Alexandrov LB Martincorena I . Heterogeneity of genomic evolution and mutational profiles in multiple myeloma. Nat Commun (2014) 5:2997. doi: 10.1038/ncomms3997 24429703 PMC3905727

[36] Furukawa Y Kikuchi J . Molecular basis of clonal evolution in multiple myeloma. Int J Hematol (2020) 111:496–511. doi: 10.1007/s12185-020-02829-6 32026210

[37] Corre J Cleynen A Robiou du Pont S Buisson L Bolli N Attal M . Multiple myeloma clonal evolution in homogeneously treated patients. Leukemia (2018) 32:2636–47. doi: 10.1038/s41375-018-0153-6 PMC6603429 29895955

[38] Luo J Solimini NL Elledge SJ . Principles of cancer therapy: Oncogene and non-oncogene addiction. Cell (2009) 136:823–37. doi: 10.1016/j.cell.2009.02.024 PMC2894612 19269363

[39] Moscvin M Ho M Bianchi G . Overcoming drug resistance by targeting protein homeostasis in multiple myeloma. Cancer Drug Resist Alhambra Calif (2021) 4:1028–46. doi: 10.20517/cdr.2021.93 PMC8903187 35265794

[40] Thibaudeau TA . & smith, d. m. a practical review of proteasome pharmacology. Pharmacol Rev (2019) 71:170–97. doi: 10.1124/pr.117.015370 PMC6423620 30867233

[41] Fonseca R Abouzaid S Bonafede M Cai Q Parikh K Cosler L . Trends in overall survival and costs of multiple myeloma, 2000–2014. Leukemia (2017) 31:1915–21. doi: 10.1038/leu.2016.380 PMC5596206 28008176

[42] Romano A Conticello C Cavalli M Vetro C La Fauci A Parrinello NL . Immunological dysregulation in multiple myeloma microenvironment. BioMed Res Int (2014) 2014:e198539. doi: 10.1155/2014/198539 PMC4071780 25013764

[43] Galon J Bruni D . Tumor immunology and tumor evolution: Intertwined histories. Immunity (2020) 52:55–81. doi: 10.1016/j.immuni.2019.12.018 31940273

[44] Grzywa TM Sosnowska A Matryba P Rydzynska Z Jasinski M Nowis D . Myeloid cell-derived arginase in cancer immune response. Front Immunol (2020) 11. doi: 10.3389/fimmu.2020.00938 PMC7242730 32499785

[45] Lelis FJN Jaufmann J Singh A Fromm K Teschner AC Pöschel S . Myeloid-derived suppressor cells modulate b-cell responses. Immunol Lett (2017) 188:108–15. doi: 10.1016/j.imlet.2017.07.003 28687234

[46] Jaufmann J Lelis FJN Teschner AC Fromm K Rieber N Hartl D . Human monocytic myeloid-derived suppressor cells impair b-cell phenotype and function *in vitro* . Eur J Immunol (2020) 50:33–47. doi: 10.1002/eji.201948240 31557313

[47] Nakamura K Kassem S Cleynen A Chrétien M-L Guillerey C Putz EM . Dysregulated IL-18 is a key driver of immunosuppression and a possible therapeutic target in the multiple myeloma microenvironment. Cancer Cell (2018) 33:634–648.e5. doi: 10.1016/j.ccell.2018.02.007 29551594

[48] Yazdani Y Mohammadnia-Afrouzi M Yousefi M Anvari E Ghalamfarsa G Hasannia H . Myeloid-derived suppressor cells in B cell malignancies. Tumor Biol (2015) 36:7339–53. doi: 10.1007/s13277-015-4004-z 26330296

[49] Steggerda SM Bennett MK Chen J Emberley E Huang T Janes JR . Inhibition of arginase by CB-1158 blocks myeloid cell-mediated immune suppression in the tumor microenvironment. J Immunother Cancer (2017) 5:101. doi: 10.1186/s40425-017-0308-4 29254508 PMC5735564

[50] Yang E Zha J Jockel J Boise LH Thompson CB Korsmeyer SJ . Bad, a heterodimeric partner for Bcl-XL and Bcl-2, displaces Bax and promotes cell death. Cell (1995) 80(2):285–91. doi: 10.1016/0092-8674(95)90411-5 7834748

[51] Zha J Harada H Yang E Jockel J Korsmeyer SJ . Serine phosphorylation of death agonist BAD in response to survival factor results in binding to 14-3-3 not BCL-XL. Cell (1996) 87:619–28. doi: 10.1016/S0092-8674(00)81382-3 8929531

[52] Chalopin T Vallet N Theisen O Ochmann M Tiab M Godmer P . No survival improvement in patients with high-risk multiple myeloma harbouring del(17p) and/or t(4;14) over the two past decades. Br J Haematol (2021) 194:635–8. doi: 10.1111/bjh.17488 33928635

