# Supplementary figures and images for: Preclinical evidence of a direct pro-survival role of arginine deprivation in multiple myeloma
Source: Front Oncol. 2022 Sep 8;12:968208. doi: 10.3389/fonc.2022.968208 (PMC9512038; doi:10.3389/fonc.2022.968208)

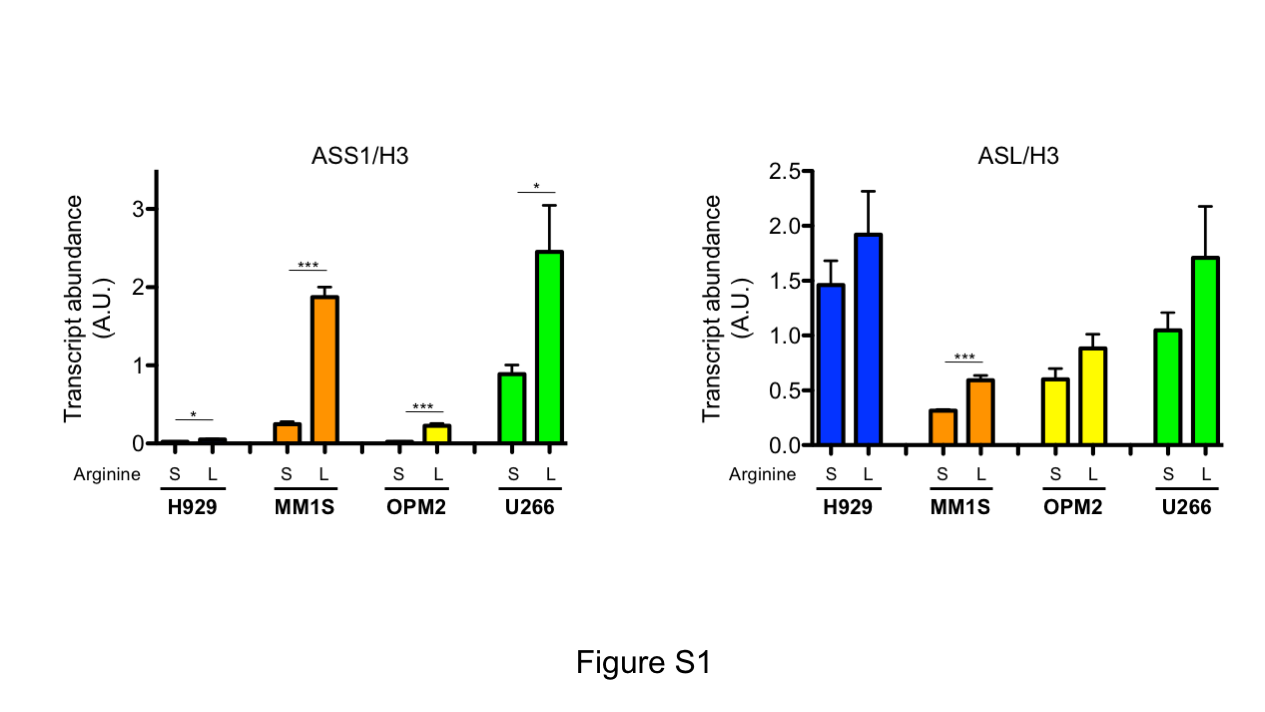

Supplement: Supplementary Figure 1 — Arginine shortage induces expression of urea cycle limiting enzymes. The indicated multiple myeloma (MM) cell lines were cultured for 24 hours (h) in complete medium containing standard (S) or low (L) arginine concentration (1140 µM or 11 µM, respectively). The histogram shows relative abundance of transcripts encoding ASS1 and ASL (real-time qRT-PCR; A.U., arbitrary unit). Bars indicate average of 4 independent experiments ± s.e.m. *p<0.05; ***p<0.001, unpaired t-test. [file Image_1.tiff]

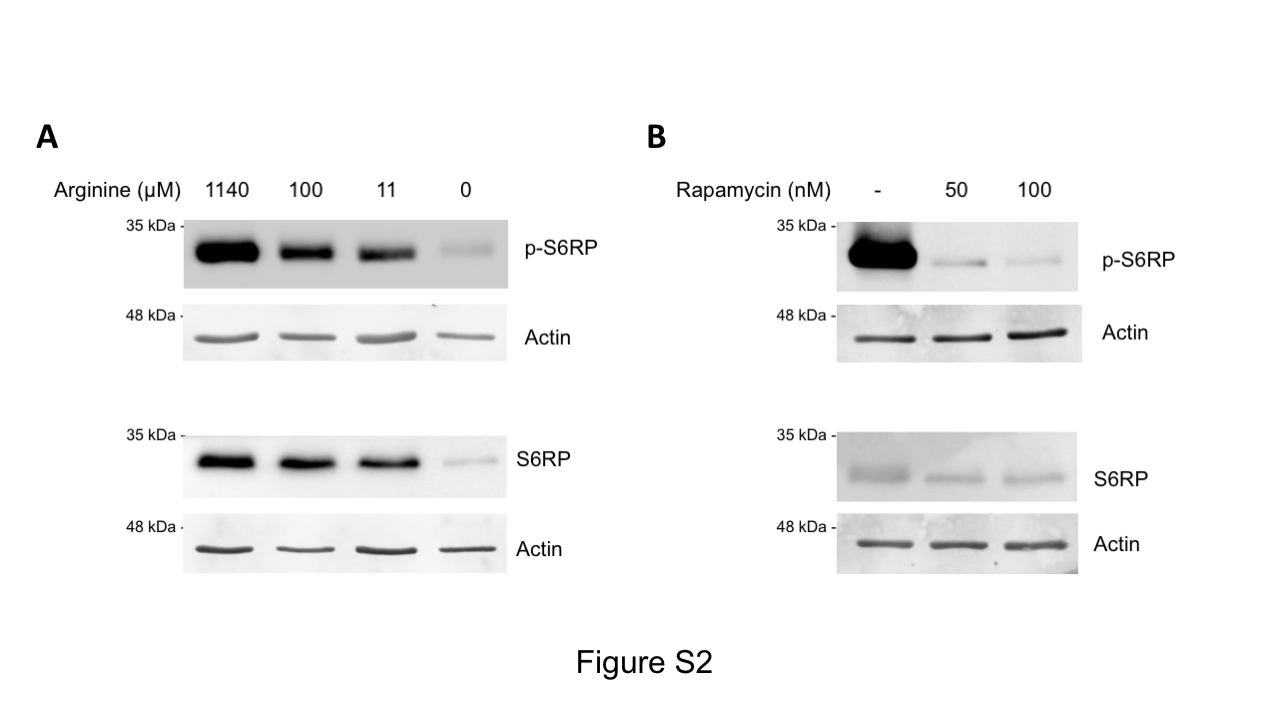

Supplement: Supplementary Figure 2 — mTORC1 is partially inhibited in cells cultured in low arginine. (A) Representative immunoblot analysis showing expression of total and phosphorylated S6RP in H929 MM cells after 24 h of culture in complete medium containing the indicated arginine concentrations (actin B serves as loading control; n≥3 independent experiments). (B) Immunoblot analysis showing the effect of 24 h treatment with the indicated doses of the established mTORC1 inhibitor, rapamycin on S6RP phosphorylation in H929 MM cells (n ≥ 3 independent experiments). [file Image_2.tiff]

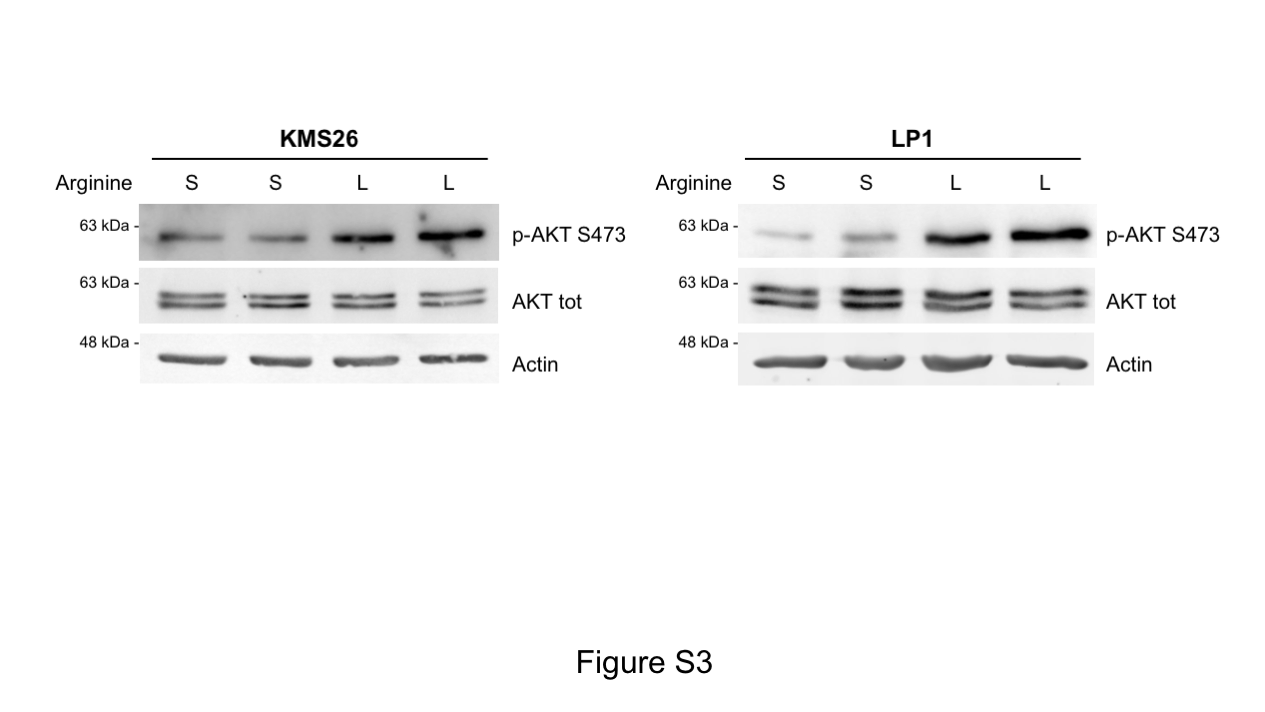

Supplement: Supplementary Figure 3 — Low arginine induces AKT phosphorylation in MM cell lines. Representative immunoblot analysis from ≥3 independent experiments showing expression of total and phosphorylated AKT at serine 473 (S473) in two independent experiments in KMS26 (left panel) or LP1 (right panel) MM cell lines after 24 h of culture in complete medium containing standard (S) or low (L) arginine concentration (1140 µM or 11 µM, respectively). Actin B serves as loading control. [file Image_3.tiff]

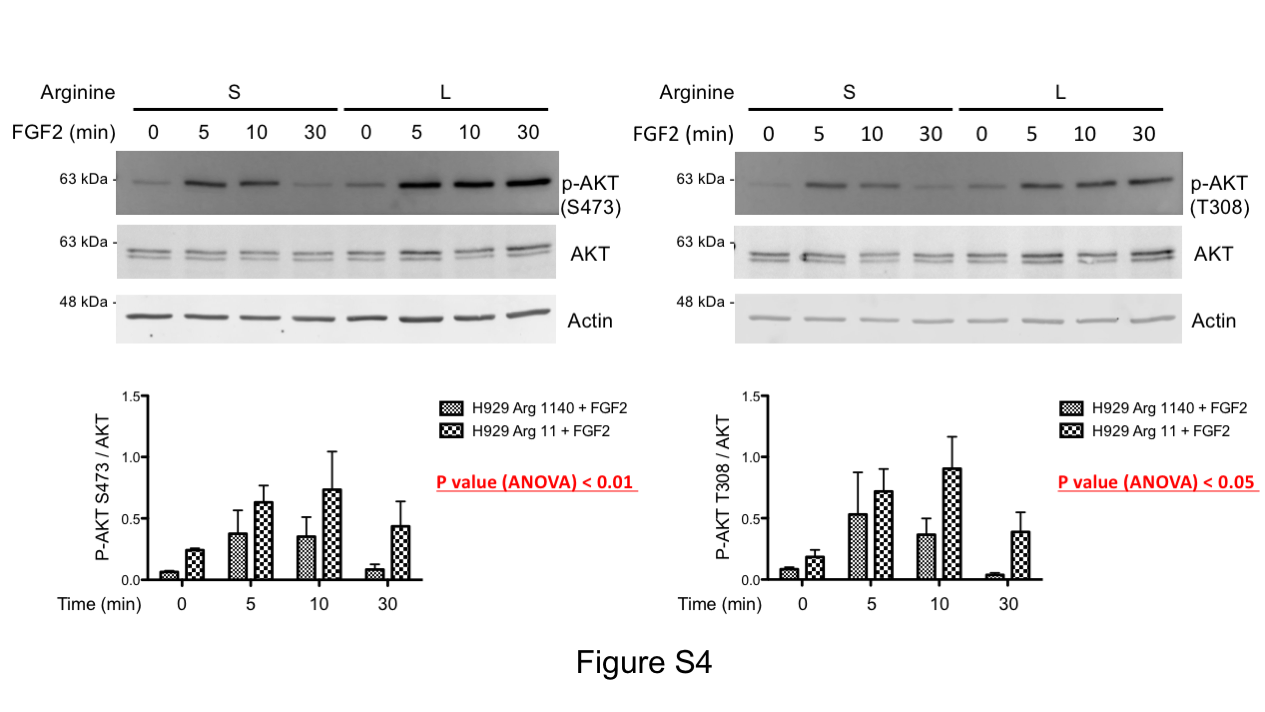

Supplement: Supplementary Figure 4 — Arginine shortage enhances FGF2-induced AKT phosphorylation. Representative immunoblot analysis from ≥3 independent experiments showing expression of total and phosphorylated AKT at serine 473 (S473) (left upper panel) and total and phosphorylated AKT at threonine 308 (T308) (right upper panel) in H929 cell line after FGF2 stimulation. Cells were cultured in medium without FBS and containing standard (S) or low (L) arginine concentration and, after 16 h, stimulated with FGF2 (10 ng/ml) for the indicated time (actin B serves as loading control). Histograms (lower panels) show quantification of phosphorylated relative to total AKT (n = 3 independent experiments) after FGF2 stimulation for the indicated time. Bars indicate average ± s.d. 2-way ANOVA. [file Image_4.tiff]

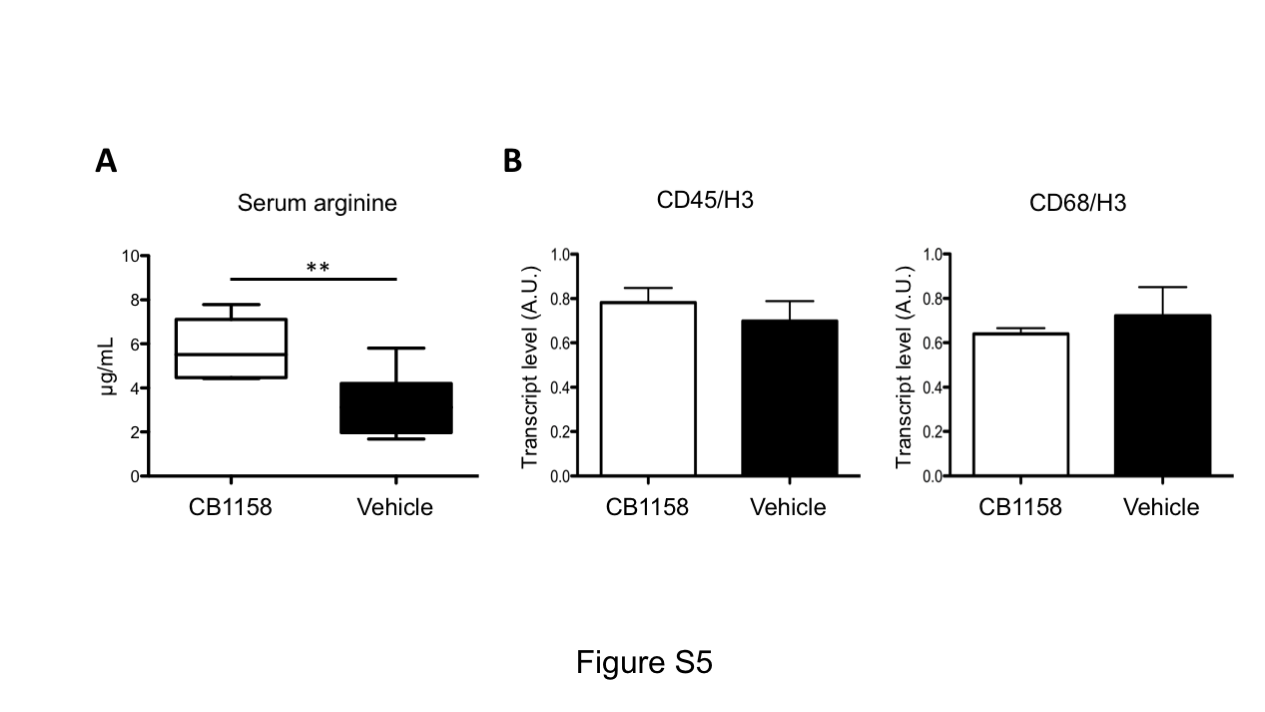

Supplement: Supplementary Figure 5 — Effect of pharmacological arginase inhibition on circulating L-arginine concentration and intratumoral expression of myelomonocytic markers in myeloma-bearing Rag–/–γc–/– mice. (A) Serum arginine concentration in tumor-bearing Rag–/–γc–/– mice treated with CB1158 or vehicle. Box and whiskers plot showing L-arginine concentration in sera from Rag–/–γc–/– mice xenotransplanted subcutaneously with H929 MM cells and treated with the arginase inhibitor CB1158 (100 µg/g of body weight) or vehicle by oral gavage bis in die for 18 days. L-arginine concentration was quantified by ELISA at the end of treatment (n = 7 mice/group). **p<0.01, unpaired t-test. (B) Relative abundance of transcripts encoding CD45 (common leukocyte marker) and CD68 (macrophage marker) in tumor biopsies at sacrifice (A.U., arbitrary units; n = 7 mice/group; average ± s.e.m.). [file Image_5.tiff]
